# Novel Van Der Waals Heterostructures Based on Borophene, Graphene-like GaN and ZnO for Nanoelectronics: A First Principles Study

**DOI:** 10.3390/ma15124084

**Published:** 2022-06-08

**Authors:** Michael M. Slepchenkov, Dmitry A. Kolosov, Olga E. Glukhova

**Affiliations:** 1 Institute of Physics, Saratov State University, Astrakhanskaya Street 83, 410012 Saratov, Russia; slepchenkovm@mail.ru (M.M.S.); kolosovda@bk.ru (D.A.K.); 2 Laboratory of Wearable Biocompatible Devices and Bionic Prostheses, I.M. Sechenov First Moscow State Medical University, Trubetskaya Street 8-2, 119991 Moscow, Russia

**Keywords:** van der Waals heterostructures, first principles calculations, density functional theory, triangulated borophene, graphene-like gallium nitride, zinc oxide, band structure, density of states, current–voltage curves

## Abstract

At present, the combination of 2D materials of different types of conductivity in the form of van der Waals heterostructures is an effective approach to designing electronic devices with desired characteristics. In this paper, we design novel van der Waals heterostructures by combing buckled triangular borophene (tr-B) and graphene-like gallium nitride (GaN) monolayers, and tr-B and zinc oxide (ZnO) monolayers together. Using ab initio methods, we theoretically predict the structural, electronic, and electrically conductive properties of tr-B/GaN and tr-B/ZnO van der Waals heterostructures. It is shown that the proposed atomic configurations of tr-B/GaN and tr-B/ZnO heterostructures are energetically stable and are characterized by a gapless band structure in contrast to the semiconductor character of GaN and ZnO monolayers. We find the phenomenon of charge transfer from tr-B to GaN and ZnO monolayers, which predetermines the key role of borophene in the formation of the features of the electronic structure of tr-B/GaN and tr-B/ZnO van der Waals heterostructures. The results of the calculation of the current–voltage (I–V) curves reveal that tr-B/GaN and tr-B/ZnO van der Waals heterostructures are characterized by the phenomenon of current anisotropy: the current along the zigzag edge of the ZnO/GaN monolayers is five times greater than along the armchair edge of these monolayers. Moreover, the heterostructures show good stability of current to temperature change at small voltage. These findings demonstrate that r-B/GaN and tr-B/ZnO vdW heterostructures are promising candidates for creating the element base of nanoelectronic devices, in particular, a conducting channel in field-effect transistors.

## 1. Introduction

Since the discovery of graphene in 2004 [1], there has been a rapid increase in the number of studies aimed at obtaining atomically thin layered 2D materials and studying their properties. Among the most discussed 2D materials, transition metal dichalcogenides (TMDC), including molybdenum disulfide and tungsten diselenide, hexagonal boron nitride (h-BN), zinc oxide (ZnO), and IV-V semiconductors should be noted [2,3,4,5,6]. The unique properties of these materials, primarily electronic and optoelectronic, make them promising candidates for replacing traditional materials in electronics, photonics, energy conversion, and storage devices [7,8,9].

In addition to studying the properties of atomically thin 2D materials and searching for their potential applications, since 2013 the attention of researchers has been attracted by the idea of forming vertical heterostructures with desired properties from individual monolayers of 2D crystals [10,11]. Due to the fact that 2D crystals in such layered structures are bound only by weak van der Waals forces, it becomes possible to combine atomically thin membranes with differences in lattice parameters inside a vertical heterostructure [12]. A wide variety of existing van der Waals heterostructures containing 2D monolayers of different types of conductivity opens the way for the development of new electronic, optoelectronic, and spintronic devices with the required functions and characteristics [13,14,15,16,17].

Vertical van der Waals heterostructures have been proposed as a platform for creating vertical transistor devices, such as tunnel field-effect transistors [18], interband tunnel transistors [19,20], barristors, and hot electron transistors [21,22]. Layer-by-layer antiferromagnetic ordering in an atomically thin crystal makes it possible to implement high-density magnetic information storage devices based on vertical van der Waals heterostructures [23]. The ultrafast and highly efficient charge transfer inherent in van der Waals heterostructures makes them a potentially promising material for photosensitive elements of optoelectronic devices. TMDC heterostructures-based solar cells are mainly formed from well-studied MoS_2_ and MoSe_2_, as well as WS_2_ and WSe_2_ [24,25,26,27,28]. Metal oxides, in particular zinc oxide and titanium dioxide, as well as third group semiconductor nitrides, such as gallium, aluminum, and indium nitride, are promising materials for creating UV radiation photodetectors [29]. Among the ways to implement UV radiation sensors, preference is given to detection devices based on photodiodes with a Schottky barrier [30,31,32,33] formed by a metal–semiconductor contact. Such contacts are predominantly fabricated on the basis of van der Waals heterostructures containing graphene. In particular, experimental and theoretical studies have been carried out for such van der Waals heterostructures as graphene/MoS_2_ [34], graphene/boron nitride [35], graphene/InSe [36], MoTe_2_/MoS_2_ [37], graphene/phosphorene, and heterostructures based on phosphorene and MoSe_2_ allotropes [38].

At the same time, the successful synthesis of borophene [39] and the subsequent theoretical and experimental studies of borophene-based heterostructures suggest that borophene, along with graphene, can be considered by specialists as one of the promising materials for nano- and optoelectronic applications [40,41,42,43,44]. Using ab initio methods, it is predicted that borophene in combination with various 2D semiconductor materials is a promising material for the formation of the Schottky barrier. Tunable Schottky barriers based on van der Waals heterostructures borophene/InSe [44], borophene/g-C_2_N [45], borophene/MoSe_2_, and borophene/WSe_2_ [46] have already been proposed.

In this work, we propose new configurations of van der Waals heterostructures based on borophene and 2D monolayers with semiconductor properties: tr-B/GaN and tr-B/ZnO. The choice of ZnO and GaN is explained by their pronounced semiconductor properties due to the presence of a noticeable energy gap, as well as by the close correspondence of the crystal lattice parameters with the crystal lattice of borophene, which makes it possible to significantly reduce the size of the calculated atomic structures. In addition, both GaN and ZnO have been successfully used in the construction of vertical heterostructures involving graphene, which have demonstrated promising electronic and optoelectronic properties [47,48,49].

## 2. Methods and Approaches

### 2.1. Atomistic Models of van der Waals Heterostructures

**Figure 1:**
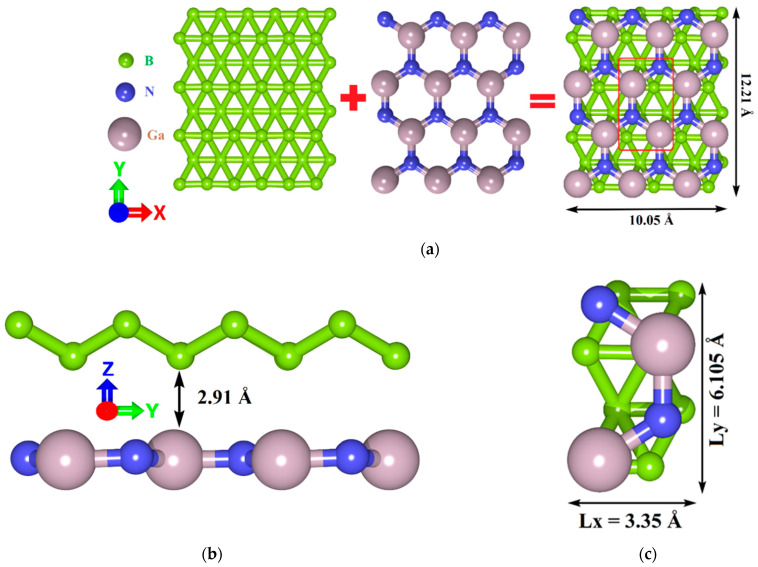
The process of constructing atomistic models of van der Waals tr-B/GaN heterostructure: top view (**a**) and side view (**b**) of an extended supercell fragment; the original supercell of tr-B/GaN heterostructure (**c**) marked with a red box in (**a**).

**Figure 2:**
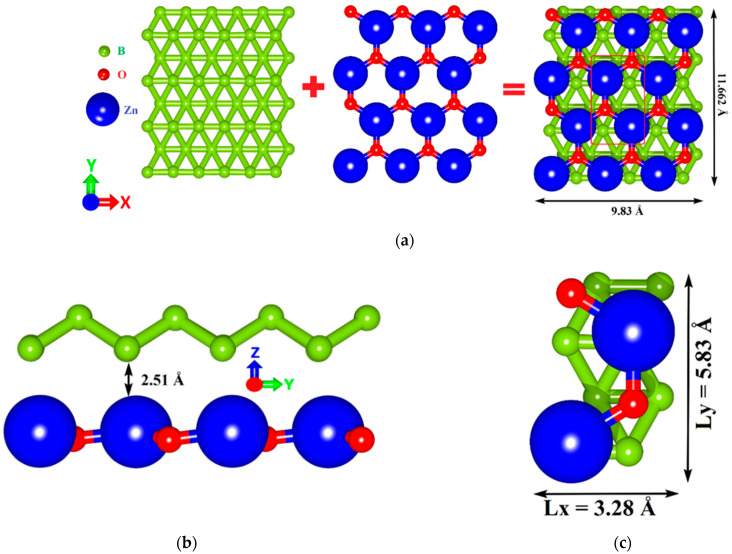
The process of constructing atomistic models of van der Waals tr-B/ZnO heterostructures: top view (**a**) and side view (**b**) of an extended supercell fragment; the original supercell of tr-B/ZnO heterostructure (**c**) marked with a red box in (**a**).

The unit cells of GaN and ZnO were taken from the open-access database Materials Project [50]. The initial translation vectors of the GaN unit cell are L_x_ = 3.204 Å and L_y_ = 5.55 Å, and the initial translation vectors of the ZnO unit cell are L_x_ = 3.205 Å and L_y_ = 5.5516 Å. Among the allotropic forms of borophene, we chose a triangulated borophene (tr-B) with high energy stability and geometric features that allow it to be combined with GaN and ZnO monolayers [51]. The unit cell of tr-B is originally set by means of atomic positions obtained from the data reported by Peng et al. [52]. The translation vectors of the tr-B unit cell are L_x_ = 1.613 Å; L_y_ = 2.864 Å. The close match between the lattice parameters of tr-B and GaN (1.613 Å (3.226 Å) and 3.204 Å, respectively, mismatch ~1%), tr-B and ZnO (1.613 Å (3.226 Å) and 3.205 Å, respectively, mismatch ~1%) makes it possible to create layered heterostructures with minimal mechanical stresses in the plane of the structure. Figure 1 and Figure 2 show the process of overlaying 2D monolayers during the formation of atomistic models of tr-B/GaN and tr-B/ZnO heterostructures with expanded rectangular cells. The original supercell of the tr-B/GaN heterostructure is shown in Figure 1c. It has optimized translation vectors L_x_ = 3.35 Å and L_y_ = 6.105 Å. The extended fragment of the tr-B/GaN supercell (see Figure 1a) was obtained by a threefold increase in the vector L_x_ and twofold increase in the vector L_y_ of the original supercell. The original supercell of the tr-B/ZnO heterostructure is shown in Figure 2c. Its translation vectors are L_x_ = 3.28 Å and L_y_ = 5.83 Å. The extended fragment of the tr-B/ZnO supercell (see Figure 2a) was obtained similarly to an extended fragment of the tr-B/GaN supercell. The distance between the tr-B and GaN monolayers along the Z-axis was 2.91 Å (see Figure 1b), between the tr-B and ZnO monolayers were 2.51 Å (see Figure 2b).

To assess the energy stability of the constructed supercells of the tr-B/GaN and tr-B/ZnO van der Waals heterostructures, we calculated the binding energy *E_b_* per atom. The calculation was carried out according to the following equation:

(1) $$E_{b}=\;\left[E_{tr-B/GaN\left(ZnO\right)}-E_{tr-B\;}-E_{GaN\left(ZnO\right)\;}\right]/N$$

where *E_tr-B/GaN(ZnO)_* is the total energy of the tr-B/GaN (tr-B/ZnO) heterostructure, *E_tr-B_* is the total energy of the isolated layer of triangulated borophene, *E_GaN(ZnO)_* is the total energy of the isolated GaN(ZnO) monolayer, *N* is the number of atoms in the heterostructure. According to the calculation results, the binding energy for a tr-B/GaN supercell is ~−0.05 eV/atom, and for a tr-B/ZnO supercell, it is ~−0.08 eV/atom. The negative value of the binding energy indicates that the tr-B/GaN and tr-B/ZnO heterostructures are energetically stable and, therefore, can be implemented in practice. In addition, in terms of energy stability (value of binding energy), they are superior to a number of other heterostructures based on GaN and ZnO monolayers, for example, MoSSe-g-GaN and WSSe-g-GaN heterostructures by almost two times [53], and ZnO/g-GeC heterostructures by more than six times [54].

### 2.2. Calculation Details

The first principle, calculations were carried out using the density functional theory (DFT) in the Siesta 4.1.5 software package [55,56,57]. The Perdew–Burke–Ernzerhof (PBE) generalized gradient approximation (GGA) [58] was used to describe exchange-correlation effects. The van der Waals interaction between heterostructure layers was described using the correction scheme proposed by Grimme [59]. When optimizing the geometry of the structure, the basis set of split valence orbitals DZP (double zeta plus polarization) was used. The Brillouin zone sampling was set as 10 × 5 × 1 Monkhorst-Pack [60] k-points mesh for all calculations. All atomic structures were fully relaxed with the force tolerance of 0.04 eV/Å. The effective Broyden–Pulay mixing scheme was used to minimize the energy of the electronic subsystem [61]. The translation vector along the z-axis of the supercell was set to be greater than 20 Å in order to avoid interaction between neighboring structures in the calculations with periodic boundary conditions. The real space mesh cutoff was chosen to be 300 Ry.

**Figure 3:**
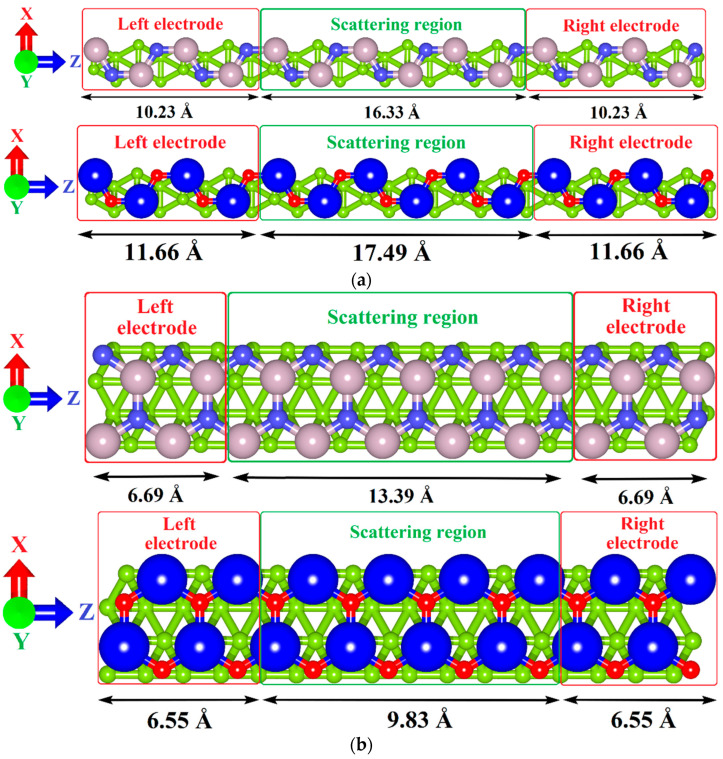
Schematic representation of a conducting channel (scattering region) enclosed between two electrodes as part of tr-B/GaN and tr-B/ZnO vertical heterostructures for current transfer along the zigzag (**a**) and armchair directions (**b**) of GaN/ZnO monolayer.

To simulate the ballistic electron transport, we used the method of nonequilibrium Green′s functions (NEGF) within the DFT framework [62,63] implemented in the TranSiesta program [55] with the DZP basis set as used. In this case, the system of electrodes and the scattering region (conducting structure) are supercells of the considered heterostructures translated along the Z-axis, as shown schematically in Figure 3. The electrodes are semi-infinite along the Z-axis and infinite along the X-axis. The scattering region is infinite along the X-axis. Two directions of current transfer were considered: along the armchair edge (Figure 3a) and along the zigzag edge (Figure 3b) of GaN/ZnO monolayer. Figure 3 also shows the dimensions of the electrodes for both directions of current transfer.

The calculation of the current through the conducting channel was carried out within the Landauer–Buttiker formula [64] using nonequilibrium Keldysh formalism [65]:

(2) $$I\left(V_{b}\right)=2e/h\int_{\mu_{R}}^{\mu_{L}}T\left(E,V_{b}\right)\;\left[f\left(E-\mu_{L}\right)-f_{2}\left(E-\mu_{R}\right)\right]dE$$

where *V_b_* is the bias voltage, *e* is the electron charge, *h* is Planck′s constant, *f*(*E*-*μ_L/R_*) are the Fermi–Dirac distribution functions for the left (*L*) and right (*R*) electrodes, respectively, *μ_L/R_* = *E_F_* ± *V_b_*/2 is the chemical potential of the left (*L*) and right (*R*) electrodes, which shifted upwards (downwards) relative to the Fermi energy *E_F_*, *T*(*E*,*V*_b_) is the quantum mechanical probability of electrons passing through the channel (transmission coefficient), which can be expressed as follows

(3) $$T\left(E,V_{b}\right)=Tr\;\left[{Г}_{L}\left(E,\;V_{b}\right)G\left(E,\;V_{b}\right){Г}_{R}\left(E,\;V_{b}\right)G^{†}\left(E,\;V_{b}\right)\right]$$

where *G*(*E*,*V*_b_), *G*^†^(*E*,*V_b_*) are the retarded and advanced Green′s functions describing the contact with the electrodes, Γ*_L_*(E), Γ*_D_*(E) are the level broadening matrices for the left (*L*) and right (*R*) electrode. Level broadening matrices for each of the electrodes are defined as

(4) $${Г}_{L/R}\left(E\right)=i\left(\Sigma_{L/R}\left(E\right)-\Sigma_{L/R}^{†}\left(E\right)\right)$$

where Σ*_L/R_* are the self-energy matrices of the left (*L*) and right (*R*) electrodes. Green′s matrices are calculated as:

(5) $$G\left(E,\;V_{b}\right)={\left(ES_{C}-H_{C}-\underset{L}{\sum }\left(E,\;V_{b}\right)-\underset{R}{\sum }\left(E,\;V_{b}\right)\right)}^{-1}$$

where *S_C_* is the overlap matrix of atomic orbitals of the conducting channel, *E* is the electron energy; *H_C_* is the Hamiltonian of the conducting channel.

## 3. Results and Discussion

### 3.1. Electronic Structure of tr-B/GaN and tr-B/ZnO Heterostructures

**Figure 4:**
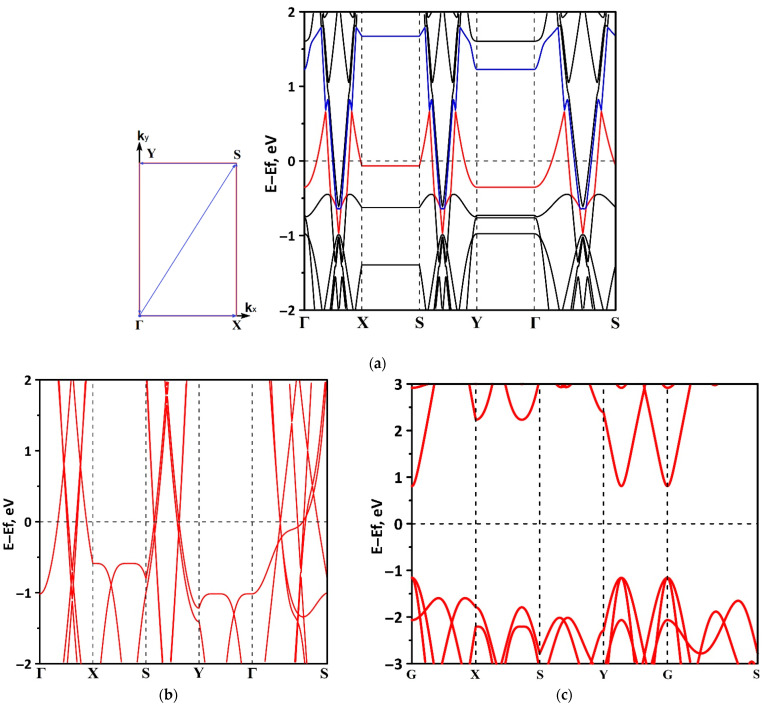
Band structure of the tr-B/GaN van der Waals heterostructure (**a**) and its constituent monolayers of triangulated borophene tr-B (**b**) and gallium nitride GaN (**c**). In (**a**), the top of the valence band is highlighted in red, and the bottom of the conduction band is highlighted in blue.

Let us turn to study the features of the electronic structure of the tr-B/GaN and tr-B/ZnO heterostructures by analyzing their band structure and DOS distribution. Note that the first Brillouin zone for the heterostructures under study is a rectangle since the supercell is periodic only in two directions. The high symmetry path for band structure within the first Brillouin zone was Γ–X–S–Y–Γ–S (see Figure 4a). The calculations were carried out on the full basis of atomic orbitals. Figure 4 shows a fragment of the band structure near the Fermi level (0 eV) for the tr-B/GaN heterostructure (Figure 4a) and for tr-B (Figure 4b) and GaN (Figure 4c) monolayers. The choice of the energy interval for representing the band structure is due to the fact that it is the electronic states at the Fermi level that make the decisive contribution to the conductive properties of the material. It can be seen that the tr-B/GaN heterostructure has a gapless band structure, in which the subbands of the valence band and conduction band touch in the direction of the wave vector *k_x_* (Г–X, S–Y, Г–S). A linear dispersion relation is observed along the Г–X, S–Y, and Г–S paths, which is characteristic of triangulated borophene (Figure 4b). Consequently, the GaN monolayer, which plays the role of a kind of substrate for borophene, does not make a decisive contribution to the electronic structure of the tr-B/GaN heterostructure. In the *k_y_* direction (X–S, Y–Г), the energy bands near the top of the valence band and the bottom of the conduction band are flat, which corresponds to a larger effective mass of charge carriers in this direction. Near the top of the valence band in Г–X, S–Y, Г–S paths, the dispersion law is close to isotropic parabolic, which is typical for the energy bands near the top of the valence band of GaN (Figure 4c). On the whole, the presence of band dispersion anisotropy in the electronic structure of tr-B/GaN should be noted.

**Figure 5:**
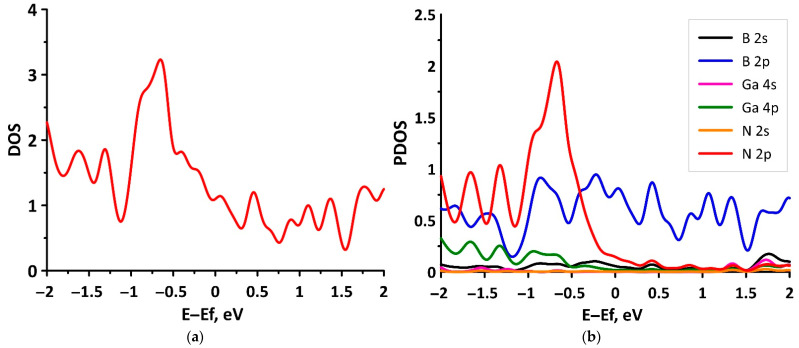
Total (**a**) and partial (**b**) DOS of the tr-B/GaN van der Waals heterostructure.

For a more detailed understanding of the features of the formation of the electronic structure of the tr-B/GaN heterostructure, Figure 5 presents the distributions of the total (DOS) and partial (PDOS) densities of electronic states. Figure 5a shows that the most intense maximum of DOS near the energy level of −0.6 eV in the valence band corresponds to the points of the Brillouin zone, where the top of the valence band (red line in Figure 4a) is in contact with the bottom of the conduction band (blue line in Figure 4a). It can be seen from Figure 5a, that the most intense maximum of DOS near the energy level of −0.6 eV in the valence band corresponds to the points of the Brillouin zone, where the top of the valence band (red line in Figure 4a) touches the bottom of the conduction band (blue line in Figure 4a). This peak of the DOS is due to the contribution of the unoccupied 2p states of nitrogen atoms (Figure 5b). In turn, the bottom of the conduction band is formed by unoccupied 2p states of boron atoms (Figure 5b), which confirms the main role of the borophene monolayer in determining the type of conductivity of the tr-B/GaN heterostructure.

**Figure 6:**
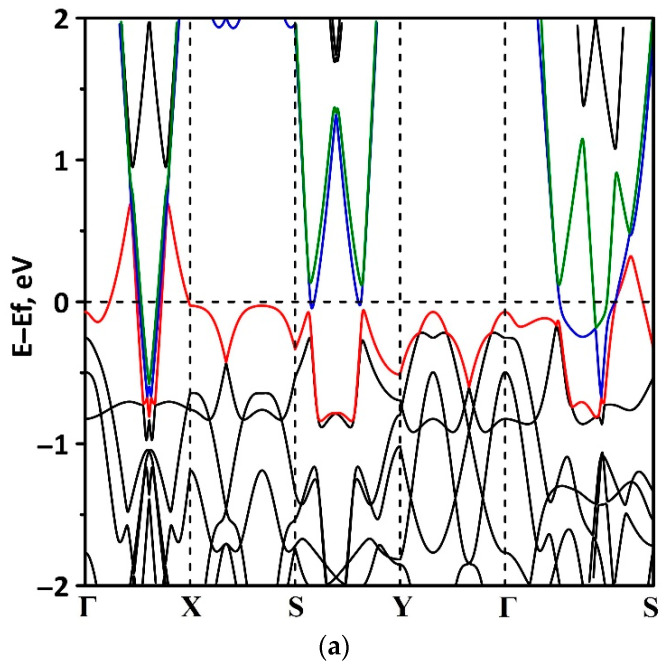

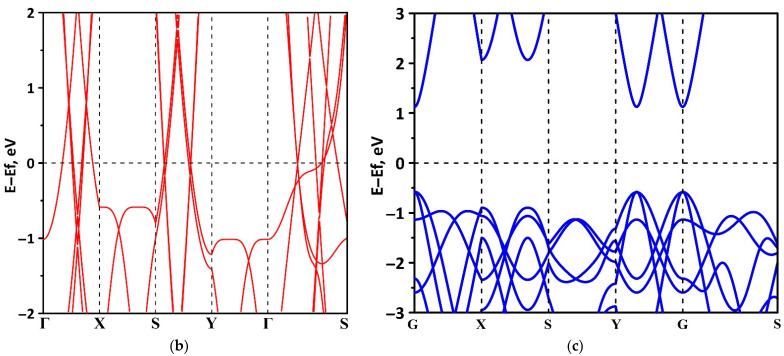
Band structure of the tr-B/ZnO van der Waals heterostructure (**a**) and its constituent monolayers of triangulated borophene tr-B (**b**) and zinc oxide ZnO (**c**). In (**a**), the top of the valence band is highlighted in red, and the bottom of the conduction band is highlighted in blue and green.

Let us turn to the consideration of the features of the electronic structure of the tr-B/ZnO heterostructure. A fragment of its band structure near the Fermi level (0 eV) is shown in Figure 6a. Fragments of the band structures of the tr-B and ZnO monolayers are shown in Figure 6b,c, respectively. As in the case of the tr-B/GaN heterostructure, the role of the triangulated borophene has become decisive in determining the conduction type of the tr-B/ZnO heterostructure. It has a gapless band structure, where the conduction band and the valence band come into contact in the G–X (in *k_x_* direction) and Г–S paths. Interestingly, the linear dispersion relation is typical for borophene in the entire *k_x_* direction (Г–X, S–Y, and Г–S, see Figure 6b), while for the tr-B/ZnO heterostructure it mainly has a place in the G–X path, both near the bottom of the conduction band and near the top of the valence band. In the S–Y and Г–S paths, the linear dispersion relation is observed only at the bottom of the conduction band, while in the valence band there is a tendency to transition from a linear dispersion relation to a parabolic one, and hence to a nonzero effective mass.

**Figure 7:**
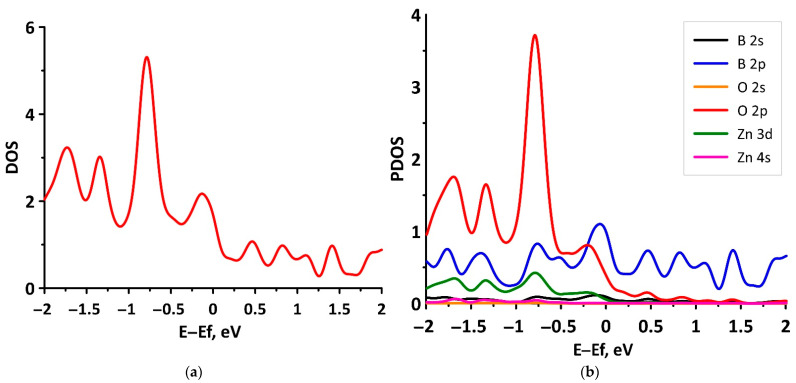
Total (**a**) and partial (**b**) DOS of the tr-B/ZnO van der Waals heterostructure.

In order to check the previously formulated conclusions about the regularities of the electronic structure of tr-B/ZnO, let us consider the graphs of the DOS and PDOS distributions presented in Figure 7. It can be seen that the most intense maximum of DOS near the energy level of –0.75 eV in the valence band corresponds to the points of the Brillouin zone, where the top of the valence band (red line in Figure 6a) touches the bottom of the conduction band (blue line in Figure 6a). This peak of the DOS is due to the contribution of the unoccupied 2p states of oxygen atoms (Figure 7b). The bottom of the conduction band is formed by unoccupied 2p states of boron atoms (Figure 7b), which confirms the conclusions about the role of borophene in determining the type of conductivity of the tr-B/ZnO heterostructure.

The key role of triangulated borophene in determining the type of conductivity of tr-B/GaN and tr-B/ZnO heterostructures can also be explained on the basis of the results of calculations of the electron charge density distribution over the atoms of their supercells according to the Mulliken procedure [66]. Based on the obtained distributions, it was revealed that as a result of the van der Waals interaction between the GaN and tr-B monolayers, as well as between the ZnO and tr-B monolayers, charge flow from borophene to GaN/ZnO is observed. In this case, the total value of the charge transferred to the GaN layer is 0.235 *e*, and to the ZnO layer, 0.22 *e*. The close values of the charge transferred for both heterostructures are explained by the proximity of Ga and Zn in the fifth row of the periodic table, and N and O in the second row of the periodic table.

### 3.2. Electrical Properties of tr-B/GaN and tr-B/ZnO Heterostructures

**Figure 8:**
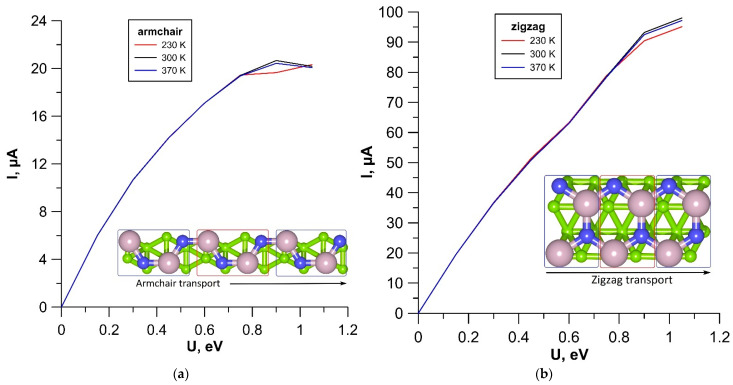
I–V curves of the tr-B/GaN heterostructure for current transfer along the armchair (**a**) and zigzag (**b**) edges of a GaN monolayer.

**Figure 9:**
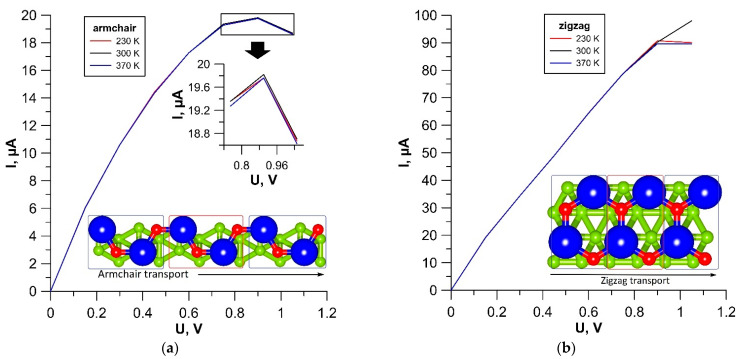
I–V curves of the tr-B/ZnO heterostructure for current transfer along the armchair (**a**) and zigzag (**b**) edges of a ZnO monolayer.

In order to assess the potential prospects for the use of tr-B/GaN and tr-B/ZnO heterostructures as the element base of nanoelectronic devices, we calculated the current–voltage (I-V) curves at various temperatures: T = 300 K, corresponding to the operation of electronic devices under normal conditions; T = 230 K and T = 370 K, corresponding to operation of devices in extreme conditions. The calculations were carried out for two directions of current transfer. In this regard, two different variants of the supercell+electrode system were prepared for each of the heterostructures. In the first variant, the current flow along the edge with an armchair configuration of GaN and ZnO monolayers was considered. In the second variant, the current flow along the edge with a zigzag configuration of GaN and ZnO monolayers was considered. The corresponding supercell structures as a conducting channel and a system of electrodes are shown in Figure 3a,b. The results of calculations of the family of I–V curves for the tr-B/GaN heterostructure are shown in Figure 8, and for the tr-B/ZnO heterostructure, in Figure 9. Since the left and right electrodes for each of the heterostructures were the same, only the direct branches of the I–V curves are shown in Figure 8 and Figure 9.

**Figure 10:**
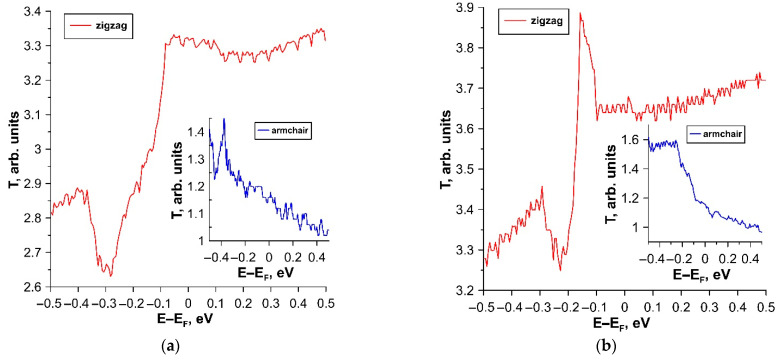
Transmission coefficient *T*(*E*) in the cases of current transfer along the zigzag and armchair directions for (**a**) tr-B/GaN and (**b**) tr-B/ZnO heterostructures.

According to the calculation results, the tr-B/GaN and tr-B/ZnO heterostructures are characterized by anisotropy of the electrically conductive properties: the current in the zigzag direction is five times greater than in the armchair direction. At the same time, both in the zigzag and in the armchair direction, the dependence of current on voltage is close to linear throughout almost the entire voltage range under consideration. Significant deviations from the linear dependence are observed at voltage values starting from 0.8–0.9 V. It should also be noted that the current values of both heterostructures are highly stable at extreme temperatures, the effect of which begins to affect only at voltages above 0.8 V. The tr-B/GaN heterostructure demonstrates the highest stability. It has a current deviation value in both directions of current transfer of no more than three μA at different temperatures. To explain the reason for the appearance of current anisotropy, let us turn to the graphs of the transmission coefficient *T*(*E*), plotted for tr-B/GaN and tr-B/ZnO heterostructures based on the calculation results. These graphs are shown in Figure 10.

It can be seen from the graphs of *T*(*E*) that the current anisotropy is explained by the fact that the number of conduction channels at the Fermi level (0 eV) in the armchair direction is several times less (~3) than in the zigzag direction. Therefore, the conduction channel in the direction of the zigzag edge has less resistance. It can be assumed that in real heterostructures the current will flow precisely along the path of least resistance.

We compared the current characteristics of tr-B/GaN and tr-B/ZnO van der Waals heterostructures and β_12_/MoS_2_ [46], SeMoS/SMoS, and SMoSe/SMoS [67] van der Waals heterostructures used as a Schottky contact. The comparison results showed that at the same voltages, the current values in the β_12_/MoS_2_ MoS_2_/WSe_2_/graphene, SeMoS/SMoS, and SMoSe/SMoS heterostructures are no more than a few nanoamperes, while the current values in the tr-B heterostructures/GaN and tr-B/ZnO is measured in tens of microamperes. In addition, the β_12_/MoS_2_ heterostructure in terms of binding energy is two times inferior to the tr-B/GaN heterostructure and three times inferior to the tr-B/ZnO heterostructure. Therefore, it can be predicted that tr-B/GaN and tr-B/ZnO van der Waals heterostructures can also be competitive as Schottky barriers for use in various nano- and optoelectronic devices.

## 4. Conclusions

In summary, we have carried out a prognostic study of the features of the atomic structure, and electronic and electrical properties of tr-B/GaN and tr-B/ZnO van der Waals heterostructures using ab initio methods. It has been established that the proposed atomic configurations of van der Waals heterostructures are energetically stable. An analysis of the band structure and total and partial DOS distributions made it possible to establish that the tr-B/GaN and tr-B/ZnO heterostructures are gapless semiconductors with band dispersion anisotropy. The mechanism of the appearance of conducting properties in heterostructures is explained by charge transfer from borophene to GaN and ZnO monolayers. The calculated families of I–V curves for two directions of current transfer showed the presence of anisotropy in the electrically conductive properties of heterostructures: the current along the zigzag edge of the ZnO and GaN monolayers in the composition of heterostructures is five times greater than along the armchair edge. The reason for the anisotropy is the smaller number of conduction channels at the Fermi level in the armchair direction, and hence the greater channel resistance in this direction. The observed effect of current anisotropy can be used in the implementation of field-effect transistors with a conducting channel based on tr-B/GaN and tr-B/ZnO van der Waals heterostructures. Considering the fact that the I–V characteristic curves of heterostructures are highly stable at extreme temperatures (230 K and 370 K), it can be predicted that in the future they can become the basis for the element base of modern nanoelectronics. In particular, they may have potential promising applications as Schottky contacts in nano- and optoelectronic devices.

## Data Availability

Not applicable.
